# Depth Influences Symbiodiniaceae Associations Among *Montastraea cavernosa* Corals on the Belize Barrier Reef

**DOI:** 10.3389/fmicb.2020.00518

**Published:** 2020-04-09

**Authors:** Ryan J. Eckert, Ashley M. Reaume, Alexis B. Sturm, Michael S. Studivan, Joshua D. Voss

**Affiliations:** Harbor Branch Oceanographic Institute, Florida Atlantic University, Boca Raton, FL, United States

**Keywords:** mesophotic coral ecosystems, dinoflagellate, amplicon sequencing, *ITS2*, symbiosis, *Cladocopium*

## Abstract

In Belize, shallow populations (10 and 16 m) of the coral species *Montastraea cavernosa* from the back reef and reef crest are genetically differentiated from deeper populations on the fore reef and reef wall (25 and 35 m). Like many species of scleractinian corals, *M. cavernosa* has an obligate symbiosis with dinoflagellate microalgae from the family Symbiodiniaceae. Here, we describe the Symbiodiniaceae taxa found within previously sampled and genotyped *M. cavernosa* populations along a depth gradient on the Belize Barrier Reef by implementing high-throughput sequencing of the *ITS2* region of Symbiodiniaceae ribosomal DNA and the *SymPortal* analysis framework. While Symbiodiniaceae *ITS2* type profiles across all sampling depths were almost entirely (99.99%) from the genus *Cladocopium* (formerly *Symbiodinium* Clade C), shallow (10 and 16 m) populations had a greater diversity of *ITS2* type profiles in comparison to deeper (25 and 35 m) populations. Permutational multivariate analysis of variance (PERMANOVA) confirmed significant differences in *ITS2* type profiles between shallow and deep sample populations. Overall Symbiodiniaceae communities changed significantly with depth, following patterns similar to the coral host’s population genetic structure. Though physiological differences among species in the cosmopolitan genus *Cladocopium* are not well-described, our results suggest that although some members of *Cladocopium* are depth-generalists, shallow *M. cavernosa* populations in Belize may harbor shallow-specialized Symbiodiniaceae not found in deeper populations.

## Introduction

The association between scleractinian corals and their endosymbiotic dinoflagellate microalgae (family Symbiodiniaceae) supports the growth and persistence of important coral reef habitats worldwide (Muscatine and Cernichiari, 1969; Hatcher, 1988). Algal symbionts are sheltered and provided inorganic nitrogen, phosphorus, and carbon; consequently, as much as 95% of their photosynthetically produced saccharides are translocated to their coral host (Muscatine and Cernichiari, 1969; Muscatine et al., 1984; Rahav et al., 1989; Venn et al., 2008; Weis, 2008). This normally mutualistic symbiosis is also susceptible to breakdown when corals are exposed to external stressors, especially thermal anomalies (Goreau, 1964; Jokiel and Coles, 1977; Glynn and D’Croz, 1990; Brown et al., 1995; Glynn, 1996). Although corals are capable of heterotrophy, they are particularly reliant upon the photosynthate produced by algal symbionts to fully meet their energetic requirements. For most coral species in close association with Symbiodiniaceae, the coral host will ultimately perish if symbiosis is not re-established within a few weeks to months after bleaching (Glynn and D’Croz, 1990; Douglas, 2003).

The high diversity of algal symbiont communities within and among coral host species contributes to the intricacies of the coral-algal symbiosis. Morphometric differences observed among symbiotic dinoflagellates provided evidence of multiple genera and species within the initially described genus *Symbiodinium* (Blank and Trench, 1985). Recent work led to the revision of the former clades within the genus *Symbiodinium sensu lato* into distinct genera within the newly re-described family Symbiodiniaceae (LaJeunesse et al., 2018). These genera consist of numerous algal symbiont “types” or “strains” which align with species-level differences (LaJeunesse et al., 2012, 2018). Presently, there is still much focus on describing these species and evaluating their diverse genetic, physiological, and ecological characteristics.

Symbiodiniaceae species may exhibit varying tolerances to environmental conditions and stressors (Baker et al., 2004; LaJeunesse et al., 2010; Wham et al., 2017). For example, many species within the genus *Durusdinium* (formerly Clade D) exhibit higher thermal tolerance than most species in other genera, making them less likely to be expelled from their coral host in times of thermal anomaly (Stat and Gates, 2011; Cunning et al., 2015a; Silverstein et al., 2015). Corals may harbor different species of Symbiodiniaceae depending upon host species, geographic location, solar irradiance levels, or water depth (van Oppen et al., 2011; Bongaerts et al., 2015b; Cunning et al., 2015b, 2017). Corals often form symbioses with one Symbiodiniaceae species, yet some corals may harbor multiple Symbiodiniaceae taxa simultaneously (Thornhill et al., 2009; Silverstein et al., 2012; Baums et al., 2014; Serrano et al., 2014; Cunning et al., 2015a). These associations may also be ephemeral, with the numerically dominant Symbiodiniaceae taxa switching following environmental stress and disturbances, particularly after thermally induced coral bleaching events (Silverstein et al., 2012, 2015).

High-throughput sequencing of the internal transcribed spacer 2 (*ITS2*) region of the ribosomal DNA operon is one of the most useful molecular methods for describing Symbiodiniaceae communities within corals (LaJeunesse, 2001; Baker, 2003; Correa and Baker, 2009; Arif et al., 2014; LaJeunesse et al., 2018). *ITS2* sequencing has been implemented to characterize Symbiodiniaceae community structure within and among coral colonies and to identify community profile shifts across environmental gradients, habitats, and temporal scales (Stat et al., 2009; Quigley et al., 2014; Klepac et al., 2015; Cunning et al., 2017; Polinski and Voss, 2018). While the *ITS2* marker is widely used in studies characterizing Symbiodiniaceae communities and provides comparisons among studies, *ITS2* is known to be multicopy and it is unclear how copy number varies among Symbiodiniaceae species (Thornhill et al., 2007). This can impact interpretations of inter- and intragenomic variation in bioinformatic analyses (Thornhill et al., 2007; Sampayo et al., 2009). Many studies collapse sequences into operational taxonomic units at 97% similarity threshold, as is common with many prokaryotic *16S* amplicon analyses (Klepac et al., 2015; Cunning et al., 2017; Kenkel and Bay, 2018). This approach can be confounded by the intragenomic variation of Symbiodiniaceae *ITS2* leading to an inability to resolve biologically relevant taxa (Smith et al., 2017; Hume et al., 2019). To overcome these hurdles, other studies have used additional markers (e.g., *psbA*^*ncr*^) in conjunction with *ITS2*, allowing more robust analysis and interpretation of *in hospite* Symbiodiniaceae (LaJeunesse and Thornhill, 2011). Hume et al. (2019) recently developed and validated the *SymPortal* analysis framework to deal with issues of resolving Symbiodiniaceae taxa based only on *ITS2* sequences. *SymPortal* identifies defining intragenomic variants (DIVs) within samples of *ITS2* sequencing data. Combinations of DIVs are then used to determine *ITS2* type profiles which are representative of putative Symbiodiniaceae taxa. This approach achieves finer resolution of inter- and intragenomic variation of Symbiodiniaceae *ITS2* without the use of additional markers (Hume et al., 2019).

Coral reefs globally are imperiled by a number of anthropogenic influences, most notably climate change (Hughes, 1994; Glynn, 1996; Gardner et al., 2003; Mumby et al., 2006; De’ath et al., 2012). Sea surface temperature models and future emission scenarios project that the majority of the world’s coral reefs will experience harmfully frequent thermal stress events in the coming decades (Hoegh-Guldberg et al., 2008; Donner, 2009; Eakin et al., 2010; van Hooidonk et al., 2016; Hughes et al., 2017; Skirving et al., 2019), which may have devastating consequences for the delicate mutualism between corals and Symbiodiniaceae and the ecosystems it supports. With the present deterioration of coral reefs and the continued threat of decline, there has been an increased focus on mesophotic coral ecosystems (MCEs; Lesser et al., 2009, 2018; Bongaerts et al., 2010). Located at 30–150 m depths, MCEs experience different thermal regimes, light spectra, and irradiance as compared to shallow coral ecosystems (Lesser et al., 2000, 2009; Leichter et al., 2006; Kahng et al., 2010; Smith et al., 2016). Despite these differences, many scleractinian species in the Tropical Western Atlantic (TWA) co-occur on both shallow reefs and MCEs (as much as 25–40%; Bongaerts et al., 2010, 2017). Due in part to the species overlap with shallow reefs, MCEs are hypothesized to be potential refuges for shallow reefs (i.e., the Deep Reef Refugia Hypothesis; Glynn, 1996; Bongaerts et al., 2010; Lesser et al., 2018; Bongaerts and Smith, 2019). Multiple studies have examined the potential for “reseeding” of shallow reefs with larvae from MCE coral counterparts using molecular methods to quantify levels of genetic connectivity between these habitats (Brazeau et al., 2013; Bongaerts et al., 2017; Studivan and Voss, 2018; Eckert et al., 2019). It is important to evaluate symbiont community assemblages as well as coral genetic structure when assessing connectivity of shallow and mesophotic reefs to give insight into the potential barriers to vertical connectivity for depth-generalist scleractinians (Bongaerts et al., 2010).

**FIGURE 1:**
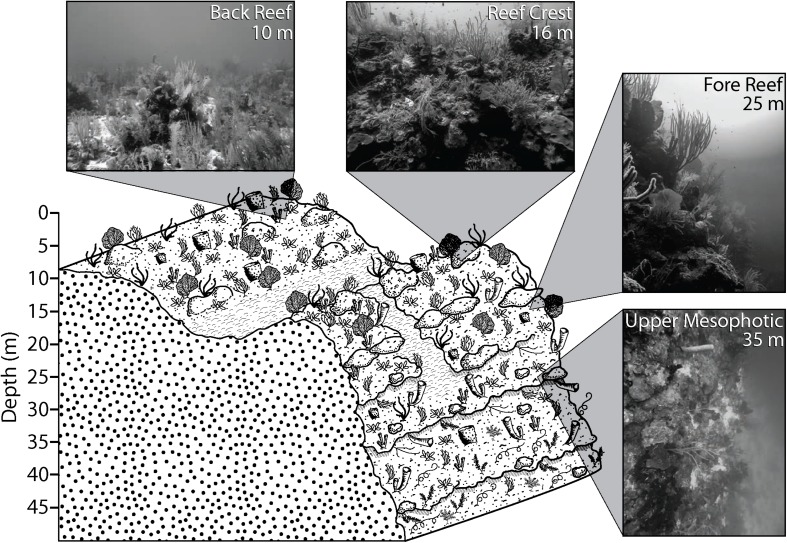
Generalized reef structure and sampling depths on the Belize Barrier Reef. Inset photographs are representative of typical habitat at sampling depths. Figure from Eckert et al. (2019).

Several studies have examined Symbiodiniaceae associated with scleractinian corals in Belize (Warner et al., 2006; Finney et al., 2010; Baumann et al., 2018), yet none have assessed how Symbiodiniaceae vary across shallow and mesophotic depths. The Belize Barrier Reef surrounding Carrie Bow Cay and the seaward margin of Glover’s Reef Atoll provide abundant mesophotic habitat directly adjacent to shallow coral ecosystems. These reefs exhibit spur and groove structures to a depth of 20–33 m, a near-vertical step from 30–37 m, and a sloping reef wall continuing to >100 m (James and Ginsburg, 1979; Figure 1). Populations of the depth-generalist, broadcast spawning, scleractinian coral *Montastraea cavernosa* lack gene flow between relatively shallow (10 and 16 m) and deep (25 and 35 m) populations within the South Water Caye and Glover’s Reef Marine Reserves (Eckert et al., 2019). To better understand if the identity of algal endosymbionts associated with *M. cavernosa* populations in Belize followed similar patterns as observed in genetic structuring of their coral hosts, we characterized the Symbiodiniaceae found within previously genotyped *M. cavernosa* samples using the high-throughput sequencing of the *ITS2* marker and the *SymPortal* analysis framework.

## Materials and Methods

### Sampling Sites and Sample Collection

**FIGURE 2:**
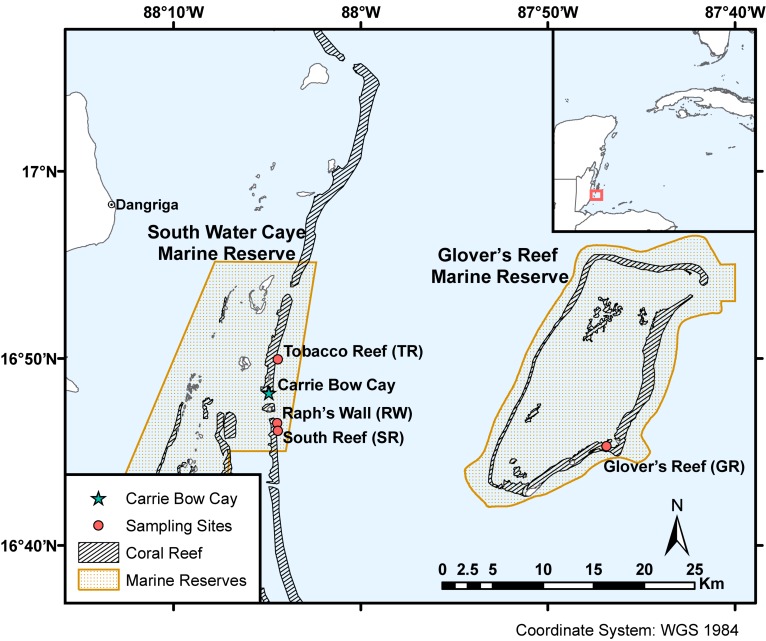
Sampling sites within South Water Caye and Glover’s Reef Marine Reserves on the Belize Barrier Reef. Coral reef habitat and MPA shapefiles adapted from Meerman and Clabaugh (2017).

This study examined the algal endosymbionts within *M. cavernosa* populations found across two marine reserves (South Water Caye and Glover’s Reef Marine Reserves) on the Belize Barrier Reef. Samples were collected from three sites along the barrier reef near Carrie Bow Cay (South Reef, Raph’s Wall, and Tobacco Reef) and one site on Glover’s Reef Atoll (Glover’s Reef), ∼30 km southeast of Carrie Bow Cay (Figure 2). Samples were collected from sites containing sufficiently abundant *M. cavernosa* across all depth zones. Approximately 15 *M. cavernosa* colonies were sampled at each of the four depth zones (back reef ∼10 m; reef crest ∼16 m; fore reef ∼25 m; upper mesophotic ∼35 m; Figure 1) per reef site (*n* = 242). Sample collection and initial processing are detailed in Eckert et al. (2019). After field collection and processing at Carrie Bow Cay, samples were preserved in TRIzol reagent and initially stored at –20°C. Samples were then transported to FAU-HBOI on ice and stored at –80°C until genomic DNA extraction. Samples were collected over two field expeditions (Table 1). All samples were collected in the spring, nearly one year apart, to avoid potential seasonal Symbiodiniaceae community shifts. Samples from 35 m depth zones within South Water Caye Marine Reserve (i.e., Tobacco Reef, Raph’s Wall, and South Reef; *n* = 45) were collected in March 2016 (Studivan and Voss, 2018), and all remaining samples (*n* = 137) in South Water Caye Marine Reserve were collected in March 2017. All samples (*n* = 60) from Glover’s Reef Marine Reserve were collected in March 2017.

**TABLE 1:** Site and sampling information for *Montastraea cavernosa* samples collected near Carrie Bow Cay, Belize.

**Site name**	**Latitude**	**Longitude**	**Depth zone**	***n***	**Sampling date**
Tobacco Reef (TR)	16°49.946′ N	88°4.441′ W	10 m	15	25 March 2017
			16 m	15	24–25 March 2017
			25 m	15	25 March 2017
			35 m	15	7–8 March 2016
Raph’s Wall (RW)	16°46.564′ N	88°4.479′ W	10 m	15	23 March 2017
			16 m	15	23 March 2017
			25 m	16	23 March 2017
			35 m	15	6 March 2016
South Reef (SR)	16°46.137′ N	88°4.433′ W	10 m	16	24 March 2017
			16 m	15	24 March 2017
			25 m	15	24 March 2017
			35 m	15	5 and 8 March 2016
Glover’s Reef (GR)	16°45.323′ N	87°46.875′ W	10 m	15	27 March 2017
			16 m	15	27 March 2017
			25 m	15	27 March 2017
			35 m	15	27 March 2017

### Symbiodiniaceae *ITS2* Amplicon Sequencing Library Preparation

Total genomic DNA was extracted using a modified cetyl trimethylammonium bromide (CTAB) extraction (Mieog et al., 2009) as in Eckert et al. (2019). Following DNA extraction, all samples were cleaned with the Zymo Research DNA Clean & Concentrator-5 kit to enhance downstream polymerase chain reaction (PCR) amplification. Cleaned extracts were checked for quality and concentration with a NanoDrop 2000 (Thermo Fisher Scientific) spectrophotometer and sample dilutions were prepared for PCR amplifications.

The *ITS2* region of Symbiodiniaceae ribosomal DNA operon was targeted for sequencing using Symbiodiniaceae specific primers *its-dino* and *its2rev2* (Pochon et al., 2001; Stat et al., 2009) modified to include adapter regions for the incorporation of indexed forward and reverse Illumina adapters (Klepac et al., 2015; Supplementary Table S1). Each 30 μL PCR included the following components: 1U Takara HS Taq, 1X Takara Taq Buffer, 0.15 μM each forward and reverse primer, 0.25 mM dNTP mixture, and 20 ng of template genomic holobiont DNA. All PCRs were run with an initial melt of 95°C for 5 min, followed by 22–28 cycles of 95°C for 40 s, 65°C for 2 min, and 72°C for 1 min, and a final extension of 72°C for 10 min. To avoid over-amplification, any samples with only a faint band visible on a 2% agarose gel after 22 cycles received an additional 1–6 PCR cycles following the same PCR profile without the initial melt of 95°C for 5 min (Kenkel et al., 2013; Klepac et al., 2015). Samples that did not amplify after 28 cycles were excluded from further analyses (*n* = 1; Supplementary Table S2).

PCR products were cleaned with the Thermo Scientific GeneJET PCR Purification Kit according to manufacturer protocols, quantified fluorescently using Qubit (Invitrogen), and diluted for subsequent PCRs. Samples were randomly assigned to one of three sequencing pools (∼80 samples per pool) and a second PCR was run on each sample to incorporate a unique combination of indexed forward and reverse Illumina adapter primers producing a unique dual index (i.e., “barcode”) for each sample in each pool (Klepac et al., 2015; Supplementary Table S1). A 20 μL PCR was run for each sample in each pool with 15 ng initial PCR product and 0.15 μM of each indexed Illumina forward and reverse adapter primer. All other components were identical to the initial *ITS2* amplification PCR. Cycling conditions were identical to initial PCRs, but with only 4 cycles required to incorporate indexed adapters.

Indexed PCR products were run on a 2% agarose gel with SYBR Green (Invitrogen). The resulting ∼500 bp amplicon was excised, extracted with the QIAGEN Gel Extraction Kit, and quantified using quantitative PCR (qPCR) on an Eppendorf Realplex4 using Thermo Scientific Maxima SYBR Green qPCR Master Mix with 0.1 μM Illumina adapter primers (Supplementary Table S1). Indexed *ITS2* libraries were pooled based on calculated cycling thresholds (C_*T*_) to ensure equitable representation among samples in each sequencing pool. Pooled libraries were purified and concentrated through isopropanol precipitation and eluted in nuclease-free water for sequencing. The libraries were loaded and sequenced with 20% *phiX* on the Illumina MiSeq platform (v3 chemistry) using paired-end 300 bp reads.

### Amplicon Sequencing Analysis With *SymPortal*

Demultiplexed forward and reverse. fastq. gz files were remotely submitted to SymPortal.org for analysis and were subjected to standard sequence quality control protocols implemented with MOTHUR 1.39.5 (Schloss et al., 2009), the BLAST+ suite of executables (Camacho et al., 2009), and minimum entropy decomposition (Eren et al., 2015) to filter non-Symbiodiniaceae and sequencing artifacts from the dataset (Hume et al., 2019). Sequences were grouped by genera and only groups with more than 200 sequences were algorithmically searched. Sequences occurring in a sufficient number of samples within both the dataset being analyzed and the entire database of samples run through *SymPortal* were identified as DIVs which were then used to characterize *ITS2* type profiles (Hume et al., 2019).

### Statistical Analysis of *SymPortal* Results

Subsequent statistical analyses of Symbiodiniaceae diversity were conducted on *SymPortal* outputs in the *R* statistical environment (R Core Team, 2019) and PRIMER v7 software package (Clarke and Gorley, 2015). To account for differences in sequencing depth among individual libraries, resulting *ITS2* sequences and *ITS2* type profile reads were normalized using trimmed mean of *M*-values (TMM) in the package *edgeR* (Robinson and Oshlack, 2010), which effectively decreases false discovery rates and increases true positive rates (Pereira et al., 2018). Non-metric multidimensional scaling analyses were conducted in PRIMER v7 using Bray-Curtis dissimilarities of square root-transformed sample read counts to visualize differences in beta diversity of *ITS2* sequences and *ITS2* type profiles.

Subsequent statistical analyses were carried out on *ITS2* type profile sequencing reads, which are representative of putative Symbiodiniaceae taxa. The *betadisper* function was used in the package *vegan* to calculate multivariate homogeneity of dispersion (PERMDISP) using Bray-Curtis distances (Oksanen et al., 2019). Pairwise comparisons were calculated with permutation tests using the *permutest* function in *vegan* for any significant factors (9,999 permutations). Permutational multivariate analysis of variance (PERMANOVA) was used to test for differences in Symbiodiniaceae *ITS2* type profiles, due to the balance of sampling design and the demonstrated lack of sensitivity to heterogeneity of dispersion compared to other multivariate statistical tests (e.g., ANOSIM; Anderson and Walsh, 2013). Depth and sampling site were used as fixed factors in the *adonis* function in *vegan* with 9,999 permutations of residuals from Bray-Curtis dissimilarities. After significant PERMANOVA results, pairwise PERMANOVA tests were conducted with the package *pairwiseAdonis* (Martinez Arbizu, 2017) using false discovery rate (FDR) corrected *p*-values. PERMANOVA were also run on a subset of the data with 35 m samples removed to test if different sampling times (2016 vs. 2017) influenced the observed results. Finally, similarity percentage (SIMPER) tests were run in PRIMER v7 using an 80% cumulative similarity cutoff to determine which *ITS2* type profiles contributed most to significant differences among factors identified by pairwise PERMANOVA.

## Results

### Symbiodiniaceae *ITS2* Sequences and *ITS2* Type Profiles

**FIGURE 3:**
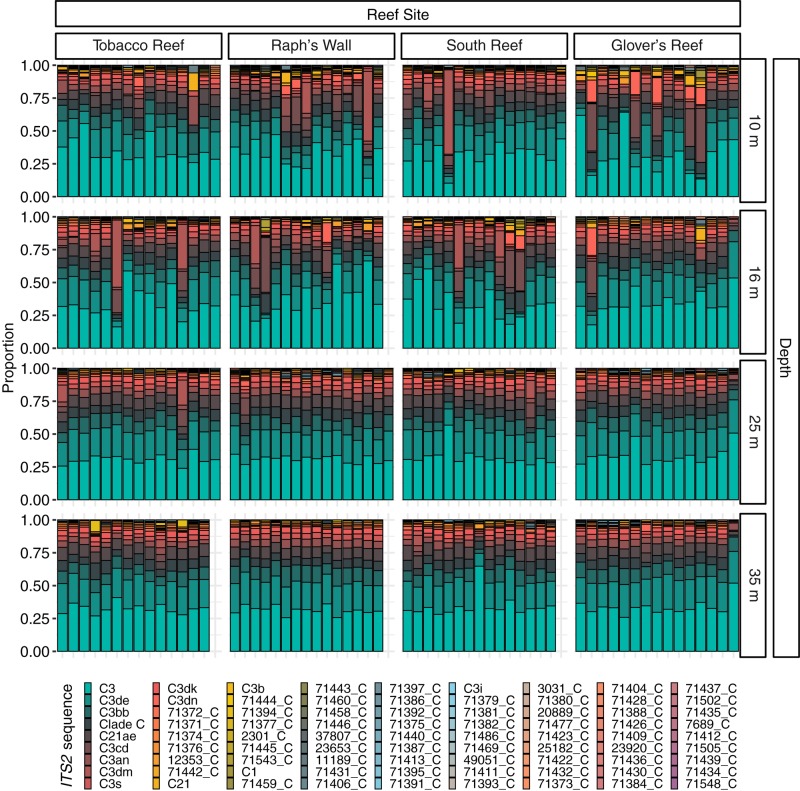
Normalized relative proportion of *ITS2* sequences from *Montastraea cavernosa* samples. Only the most abundant (>0.01% of all reads) sequences are displayed (*n* = 81). *ITS2* sequences are listed in order of overall abundance. Sequences not used in the definition of *ITS2* type profiles are named with a unique database ID number followed by a letter which refers to the genus the sequence is from (e.g., 71372_C is a sequence from the genus *Cladocopium* with the database ID 71372).

**FIGURE 4:**
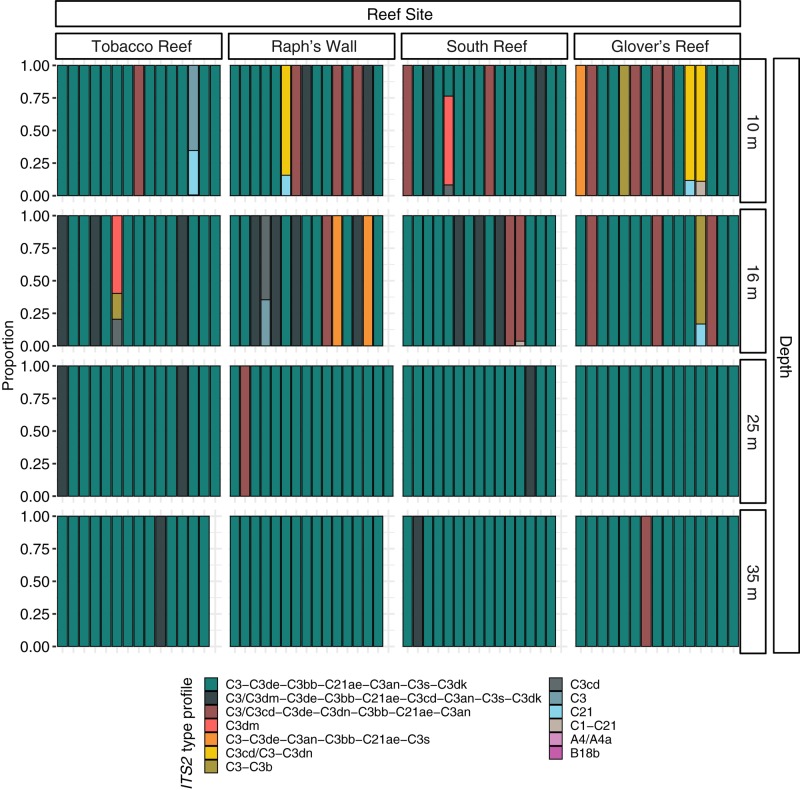
Normalized relative proportion of *ITS2* type profiles from *Montastraea cavernosa* samples. *ITS2* type profiles are listed in order of overall abundance. *ITS2* type profiles are named for the defining intragenomic variants (DIVs) used to characterize them. DIVs in a type profile name are listed in order of abundance with ‘/’ separating DIVs with co-dominance in samples.

Prior to quality filtering, the 241 samples returned 46,476,815 sequencing reads, 30,858,017 of which passed the described initial quality and sample assignment filters (66.39%). *ITS2* sequences from the genera *Symbiodinium* (formerly Clade A), *Breviolum* (formerly Clade B), and *Cladocopium* (formerly Clade C) were used to calculate *ITS2* type profiles, with the majority of filtered *ITS2* sequences being of the genus *Cladocopium* (99.99%; Figure 3). Thirteen *ITS2* type profiles were identified across all samples, eleven of which were from the genus *Cladocopium*, with the remaining *Symbiodinium* and *Breviolum* profiles comprising 0.00017% of all *ITS2* type profiles (Figure 4).

### Symbiodiniaceae Variation Across Depth

**FIGURE 5:**
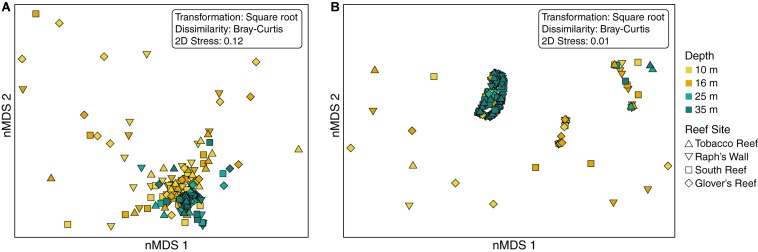
Non-metric multidimensional scaling (nMDS) biplot based on **(A)** *ITS2* sequences and **(B)** *ITS2* type profiles using Bray-Curtis dissimilarities. Each point represents the Symbiodiniaceae found within a *Montastraea cavernosa* sample. Color denotes sampling depth and shape denotes reef site.

Visualization of beta diversity of *ITS2* sequences with nMDS revealed that most samples from 25 and 35 m populations, hereafter referred to as “deep populations,” clustered together while samples from 10 and 16 m, hereafter referred to as “shallow populations,” were much less tightly clustered (Figure 5A). Ordination of *ITS2* type profiles with nMDS illustrated strong clustering of the majority of samples (Figure 5B). Nearly all (94.17%) of the deep population samples grouped together in the main cluster, with shallow population samples much more dispersed in comparison (66.12% in the main cluster). Beta diversity of *ITS2* type profiles was significantly higher in shallow populations (*F* = 11.565_3_, _237_, *p* < 0.0001; Table 2) than deep populations but did not differ significantly across sampling sites.

**TABLE 2:** Test results for homogeneity of multivariate dispersions (PERMDISP) and pairwise permutation tests using the *betadisper* and *permutest* functions in *vegan*.

**Test**	**Comparison**	**Test statistic**	***p-*value**
PERMDISP	Site	0.5837	ns
	Depth	11.565	0.0001
Permutation test	10 m vs. 16 m	–0.1384	ns
	10 m vs. 25 m	3.9797	0.0004
	10 m vs. 35 m	4.2363	0.0001
	16 m vs. 25 m	4.0923	0.0001
	16 m vs. 35 m	4.3452	0.0001
	25 m vs. 35 m	0.3414	ns

*Pairwise *p*-values are the permuted *p-*values generated by the test (9,999 permutations). Non-significant tests are listed as “ns.”*

PERMANOVA results indicated that Symbiodiniaceae communities varied significantly by depth (Pseudo-*F* = 6.880_3_, _225_, *p* = 0.0001), but that neither site nor the interaction between site and depth had any significant effects on community composition. Pairwise PERMANOVA identified that differences in Symbiodiniaceae communities occurred between shallow populations and deep populations (Table 3). Removing all 35 m samples did not change results of PERMANOVA or pairwise comparisons, demonstrating that time differences between sampling trips did not significantly influence patterns in algal symbiont community structure (Supplementary Table S3). SIMPER tests found shallow and deep populations were 37.46% dissimilar to one another on average. The three most abundant *ITS2* type profiles (Figure 4) accounted for over 85% of the dissimilarity between shallow and deep populations (Table 4).

**TABLE 3:** Test results from permutational multivariate analysis of variance (PERMANOVA; 9,999 permutations) of Symbiodiniaceae *ITS2* type profiles from *M. cavernosa* colonies and pairwise comparisons between all depth zones (FDR corrected).

**Test**	**Comparison**	**Pseudo-*F***	***p*-value**
Overall	Depth	6.8801	0.0001
	Site	1.4559	ns
	Depth:Site	1.1611	ns
Depth	10 m vs. 16 m	0.9838	ns
	10 m vs. 25 m	9.0844	0.0005
	10 m vs. 35 m	10.0260	0.0004
	16 m vs. 25 m	9.4051	0.0004
	16 m vs. 35 m	10.6563	0.0004
	25 m vs. 35 m	0.1196	ns

*Non-significant tests are listed as “ns.”*

**TABLE 4:** Similarity percentage (SIMPER) test results.

**Group**	**Average Dissimilarity**	***ITS2* type profile**	**Contribution**
Shallow vs. Deep	37.46%	C3-C3de-C3bb-C21ae-C3an-C3s-C3dk	46.43%
		C3/C3dm-C3de-C3bb-C21ae-C3cd-C3an-C3s-C3dk	19.64%
		C3/C3cd-C3de-C3dn-C3bb-C21ae-C3an	19.13%

**ITS2* type profiles contributions to Average dissimilarity between depth zones are shown in descending order. SIMPER cutoff was set at 80%.*

## Discussion

*Cladocopium* was the most abundant algal genus identified across all *M. cavernosa* samples from all depth zones on the Belize Barrier Reef, the majority of which were C3-related sequences. These results agree with previous characterizations of *M. cavernosa* algal symbiont communities on the Belize Barrier Reef which all hosted *Cladocopium* (mainly C3 type) species as their dominant algal symbiont across a depth gradient from 8–25 m (Warner et al., 2006). The genus *Cladocopium* is one of the most species-rich, ecologically abundant, and diverse genera of Symbiodiniaceae (LaJeunesse et al., 2018). Some members of the genus, *C. goreaui* for example, are considered host-generalists because they exist in association with many coral species and across broad environmental, geographic, and depth ranges (LaJeunesse, 2001, 2005; Serrano et al., 2014; LaJeunesse et al., 2018). Previous studies across multiple regions have reported *M. cavernosa* harboring *Cladocopium* spp. almost exclusively (Warner et al., 2006; Serrano et al., 2014; Bongaerts et al., 2015b; Klepac et al., 2015; Polinski and Voss, 2018). Similar to the results presented here, *Cladocopium* spp. have also been identified as the predominant symbiont species within *M. cavernosa* on reefs in Florida, United States Virgin Islands, Barbados, Bermuda, as well as other sites in Belize (Finney et al., 2010; Serrano et al., 2014). Just as *Cladocopium* spp. are extremely cosmopolitan reef inhabitants, *M. cavernosa* is one of the most ubiquitous coral species in the TWA, potentially due in part to its association with this common genus of algal symbiont. Many species of *Cladocopium* are also found associated with coral species at depths >60 m (Lesser et al., 2010; Bongaerts et al., 2015b; Lucas et al., 2016). Members of *Cladocopium* were found to be more photochemically efficient than members of *Durusdinium*, especially in temperatures ≤24°C (Silverstein et al., 2017). This may make *Cladocopium* species more beneficial symbionts at greater depths, including in mesophotic coral ecosystems where temperatures can be lower and light is more limited (Lesser et al., 2009). Niche specialization and coincidental metabolic costs across depth have been demonstrated in the algal symbiont communities of *Seriatopora hystrix* colonies (Cooper et al., 2011). While *Durusdinium* spp. were dominant in *S. hystrix* between 3–23 m, *Cladocopium* dominated endosymbiont communities were more common between 23–45 m, coinciding with a decline in net photosynthetic production.

Forming symbiotic relationships predominantly with a cosmopolitan and hyper-diverse Symbiodiniaceae genus may be advantageous to high dispersal, allowing *M. cavernosa* to dominate and persist across most reefs in the TWA. However, some studies examining *M. cavernosa* Symbiodiniaceae communities have also detected background (≤5%) levels of other genera, including *Symbiodinium, Breviolum* and *Durusdinium* (Serrano et al., 2014; Klepac et al., 2015; Polinski and Voss, 2018). All of these genera have been previously identified within other scleractinian species on the Belize Barrier Reef (Baumann et al., 2018), but we only detected minimal abundances (<0.01%) of *ITS2* type profiles from *Symbiodinium* and *Breviolum* in a minority of our samples (*n* = 2), demonstrating an affinity for symbioses between *M. cavernosa* and *Cladocopium* spp. in this region.

Previous examination of Symbiodiniaceae from mesophotic and shallow *M. cavernosa* has found significant differences in communities and physiology across depth in multiple regions. In the northwestern Gulf of Mexico (NW GOM), *M. cavernosa* from MCEs had higher densities of Symbiodiniaceae cells and greater levels of chlorophyll *a* and chlorophyll *c*_2_ per unit area of coral tissue in comparison to shallow conspecifics (Polinski and Voss, 2018). Despite these differences, there was no significant difference among Symbiodiniaceae communities observed across depth in the NW GOM. Other studies have found differences in Symbiodiniaceae communities of *M*. *cavernosa* and other scleractinian corals across depth in the Bahamas (Lesser et al., 2010) and Curaçao (Bongaerts et al., 2015a, b). In these instances, significant differences were observed in the lower mesophotic zone (>60 m). However, in Curaçao there was also a significant shift in symbiont community profile observed at 25 m for *M. cavernosa* (Bongaerts et al., 2015a), the same depth at which we have seen differences in both symbiont communities and *M. cavernosa* genetic structure in Belize (Eckert et al., 2019). While there is evidence for Symbiodiniaceae community shifts with increasing depth, these patterns do not appear to be universal. Rather, they appear to depend upon both coral species and region (Bongaerts et al., 2010, 2015a, 2017).

Symbiodiniaceae *ITS2* type profiles from *M. cavernosa* on the Belize Barrier Reef are depth-stratified, but not as distinctly as *M*. *cavernosa* population genetic structure. The symbiont community “break point” still appears to remain between the geomorphologic transition between reef crest and fore reef (16 and 25 m; Figure 1), but rather than having distinct depth-specialized assemblages, there is a relatively abundant depth-generalist *ITS2* type profile in Belize (Figure 4). Shallow communities instead show greater algal diversity across coral samples, characterized by the presence of additional *ITS2* type profiles not present in samples beyond 16 m. In Curaçao, multiple depth-generalist corals also harbored more diverse Symbiodiniaceae communities on shallow reefs, but overall, hosted similar algal symbiont compositions between shallow and mesophotic depths, until depths of 50–60 m (Bongaerts et al., 2015a). These results are similar to what we observed in the algal symbiont communities of *M. cavernosa* across shallow and upper mesophotic depths on the Belize Barrier Reef. There may be extreme depth-specialized communities in Belize among deeper (i.e., 40–60 m) *M. cavernosa* populations not captured in this study, as reported in other regions (Lesser et al., 2010; Bongaerts et al., 2015a).

On the Belize Barrier Reef, we found the majority (94.17%) of deep populations of *M. cavernosa* were dominated by a single *ITS2* type profile with only 2 additional *ITS2* type profiles present in these populations. The majority of all sampled colonies along the depth gradient from 10–35 m had the same Symbiodiniaceae *ITS2* type profile (Figure 4; *n* = 192). Recent studies suggest that there is a high level of intra-genus diversity in physiological tolerances of algal symbionts. For example, a consensus ranking algorithm found that thermal tolerance of different *Cladocopium* species ranged highly, between the 3rd and 71st percentile (Swain et al., 2017). There is the potential that the *ITS2* type profiles which were only present in shallow populations of *M. cavernosa* are specialized for shallow reef habitats. Previous work has suggested that the taxa of *in hospite* Symbiodiniaceae in coral can be significantly influenced by the availability and diversity of free-living Symbiodiniaceae present in the surrounding environment (Cunning et al., 2015b; Quigley et al., 2017). However, there is limited information on whether depth is a significant factor in structuring communities of free-living Symbiodiniaceae or if the *in hospite* Symbiodiniaceae mirror free-living communities across depth. Distinct algal symbiont profiles may also be related to coral skeletal morphology and symbiont photochemistry. In the Gulf of Mexico, Symbiodiniaceae density and chlorophyll measurements changed with host morphology (Polinski and Voss, 2018; Studivan et al., 2019). Observed differences in Symbiodiniaceae communities found in Belize may also occur as a function of host morphology as in the Gulf of Mexico. We are presently unable to assess this hypothesis due to the small fragment sizes sampled (∼ 6 cm^2^).

While this study implemented a balanced sampling design over depth and site, sampling occurred over two excursions (Table 1). This could introduce variance over time, due to the temporal changes which may occur in the numerically dominant Symbiodiniaceae within a coral colony (Baker, 2003; Berkelmans and van Oppen, 2006; Reich et al., 2017). A total of 13 Symbiodiniaceae *ITS2* type profiles were found within *M. cavernosa* samples taken from 10 and 16 m in March 2017. There were only three *ITS2* type profiles found in 25 m samples, even though they were also sampled in March 2017 (Table 1). Additionally, the entire complement of Glover’s Reef samples was sampled during a single day (27 March 2017) and these profiles are indistinguishable from profiles at all other sites (Table 3). *Montastraea cavernosa* has previously demonstrated stability in its Symbiodiniaceae through temporal sampling of tagged colonies, even in a comparatively variable environment (Klepac et al., 2015). Finally, there were no observed coral bleaching events between sampling events, which is a common impetus for changing of dominant Symbiodiniaceae taxa (Berkelmans and van Oppen, 2006; Silverstein et al., 2015). Based on these combined factors, the data presented here likely represent differences driven by depth rather than any temporal co-factors.

Symbiodiniaceae *ITS2* type profiles that dominated deep populations of *M. cavernosa* in this study were also typically abundant in shallow populations of *M. cavernosa*. However, a subset of Symbiodiniaceae *ITS2* type profiles unique to shallow populations contributed to the significant differences in algal symbiont assemblages reported between the shallow and deep *M. cavernosa* populations. Physiological differences among putative Symbiodiniaceae species have been previously documented, particularly in terms of thermal tolerance (Hume et al., 2015; Díaz-Almeyda et al., 2017; Silverstein et al., 2017; Swain et al., 2017), which could potentially be a driver of observed depth stratification in Belize. Nonetheless, the ubiquity of the majority of Symbiodiniaceae *ITS2* type profiles across both shallow and deep zones suggest that algal symbionts are unlikely to be driving the observed lack of gene flow between shallow and deep *M. cavernosa* populations on the Belize Barrier Reef (Eckert et al., 2019). Further resolution of Symbiodiniaceae taxonomy, coupled with investigations of environmental tolerances and preferred ranges (e.g., light, temperature, etc.) among individual Symbiodiniaceae species are needed to understand the consequences of various coral-algal symbioses and how these may drive observed variations in Symbiodiniaceae assemblages across depth zones in Belize and elsewhere.

## Data Availability Statement

All protocols, including sample preparation and data analysis scripts are available in a GitHub repository (Eckert, 2020). Raw *ITS2* amplicon sequences are available in the National Center for Biotechnology Information (NCBI) Sequence Read Archive (SRA) under the Project No. PRJNA579363, Accession Nos. SAMN13109002 to SAMN13109242.

## Author Contributions

JV and RE designed the research. JV, RE, and MS collected the coral samples. AR and RE optimized the DNA extraction and clean up. AR, AS, and RE extracted the DNA. RE prepared the sequencing libraries, performed the data analyses, and created the figures. All authors contributed to the final edited manuscript prepared by RE.

## Conflict of Interest

The authors declare that the research was conducted in the absence of any commercial or financial relationships that could be construed as a potential conflict of interest.
