# Supplementary material for: Depth Influences Symbiodiniaceae Associations Among Montastraea cavernosa Corals on the Belize Barrier Reef
Source: Front Microbiol. 2020 Apr 9;11:518. doi: 10.3389/fmicb.2020.00518 (PMC7160519; doi:10.3389/fmicb.2020.00518)
Supplement: Supplementary file 1 [file Data_Sheet_1.pdf]

Depth Influences Symbiodiniaceae Associations Among *Montastraea cavernosa* Corals on the Belize Barrier Reef

Ryan J. Eckert*, Ashley M. Reaume†, Alexis B. Sturm, Michael S. Studivan† and Joshua D. Voss*

Harbor Branch Oceanographic Institute, Florida Atlantic University, Boca Raton, FL, United States

***Correspondence:** Ryan J. Eckert ryan.j.eckert@gmail.com; Joshua D. Voss jvoss2@fau.edu

†**Present address**: Ashley M. Reaume, Department of Biological Sciences, University of Central Florida, Orlando, FL, United States
Michael S. Studivan, Cooperative Institute for Marine and Atmospheric Studies, Rosenstiel School of Marine and Atmospheric Science, University of Miami, Coral Gables, FL, United States

Supplementary Materials

Supplementary Table S1. Oligonucleotide sequences used for *ITS2* typing Symbiodiniaceae communities. Unique indices in barcoding primers are bolded in the sequence and listed under "Index".

Name	Sequence (5'-3')	Index	Use
ITS2-F-miseq	TCGTCGGCAGCGTCAGATGTGTATAAGAGACAGGTGAATTGCAGAACTCCGTG	n/a	ITS2 PCR
ITS2-R-miseq	GTCTCGTGGGCTCGGAGATGTGTATAAGAGACAGCCTCCGCTTACTTATATGCTT	n/a	ITS2 PCR
Hyb_F1_i5	AATGATACGGCGACCACCGAGATCTACAC**ATCACG**TCGTCGGCAGCGTC	ATCACG	Indexing PCR
Hyb_F2_i5	AATGATACGGCGACCACCGAGATCTACAC**CGATGT**TCGTCGGCAGCGTC	CGATGT	Indexing PCR
Hyb_F3_i5	AATGATACGGCGACCACCGAGATCTACAC**TTAGGC**TCGTCGGCAGCGTC	TTAGGC	Indexing PCR
Hyb_F4_i5	AATGATACGGCGACCACCGAGATCTACAC**TGACCA**TCGTCGGCAGCGTC	TGACCA	Indexing PCR
Hyb_F5_i5	AATGATACGGCGACCACCGAGATCTACAC**ACAGTG**TCGTCGGCAGCGTC	ACAGTG	Indexing PCR
Hyb_F6_i5	AATGATACGGCGACCACCGAGATCTACAC**GCCAAT**TCGTCGGCAGCGTC	GCCAAT	Indexing PCR
Hyb_F7_i5	AATGATACGGCGACCACCGAGATCTACAC**CAGATC**TCGTCGGCAGCGTC	CAGATC	Indexing PCR
Hyb_F8_i5	AATGATACGGCGACCACCGAGATCTACAC**ACTTGA**TCGTCGGCAGCGTC	ACTTGA	Indexing PCR
Hyb_F9_i5	AATGATACGGCGACCACCGAGATCTACAC**GATCAG**TCGTCGGCAGCGTC	GATCAG	Indexing PCR
Hyb_R1_i7	CAAGCAGAAGACGGCATACGAGAT**GGCTAC**GTCTCGTGGGCTCGG	GGCTAC	Indexing PCR
Hyb_R2_i7	CAAGCAGAAGACGGCATACGAGAT**CTTGTA**GTCTCGTGGGCTCGG	CTTGTA	Indexing PCR
Hyb_R3_i7	CAAGCAGAAGACGGCATACGAGAT**AGTCAA**GTCTCGTGGGCTCGG	AGTCAA	Indexing PCR
Hyb_R4_i7	CAAGCAGAAGACGGCATACGAGAT**AGTTCC**GTCTCGTGGGCTCGG	AGTTCC	Indexing PCR

Hyb_R5_i7	CAAGCAGAAGACGGCATACGAGAT**ATGTCA**GTCTCGTGGGCTCGG	ATGTCA	Indexing PCR
Hyb_R6_i7	CAAGCAGAAGACGGCATACGAGAT**CCGTCC**GTCTCGTGGGCTCGG	CCGTCC	Indexing PCR
Hyb_R7_i7	CAAGCAGAAGACGGCATACGAGAT**GTAGAG**GTCTCGTGGGCTCGG	GTAGAG	Indexing PCR
Hyb_R8_i7	CAAGCAGAAGACGGCATACGAGAT**GTCCGC**GTCTCGTGGGCTCGG	GTCCGC	Indexing PCR
Hyb_R9_i7	CAAGCAGAAGACGGCATACGAGAT**GTGAAA**GTCTCGTGGGCTCGG	GTGAAA	Indexing PCR
Hyb_R10_i7	CAAGCAGAAGACGGCATACGAGAT**GTGGCC**GTCTCGTGGGCTCGG	GTGCCC	Indexing PCR
Ill_Lib1	AATGATACGGCGACCACCGA	n/a	qPCR
Ill_Lib2	CAAGCAGAAGACGGCATACGA	n/a	qPCR

Supplementary Table S2. *Montastraea cavernosa* Symbiodiniaceae *ITS2* sequencing information. Site = sampling site (Fig 1.1); Depth zone = depth zone colony from which colony was sampled (Fig 1.2); *ITS2* PCR cycles = number of PCR cycles to amplify Symbiodiniaceae *ITS2*; Sequencing pool = random sequencing pool assignment; i5 index = forward index; i7 index = reverse complement of reverse index.

**Sample ID**	**Sampling date**	**Site**	**Depth zone (m)**	***ITS2* PCR cycles**	**Sequencing pool**	**i5 Index**	**i7 Index**
CBC_MCAV_001	03/23/17	RW	25	24	3	CAGATC	GCGGAC
CBC_MCAV_002	03/23/17	RW	25	24	1	CGATGT	GGAACT
CBC_MCAV_003	03/23/17	RW	25	24	2	ACTTGA	TTTCAC
CBC_MCAV_004	03/23/17	RW	25	24	1	CGATGT	TTGACT
CBC_MCAV_005	03/23/17	RW	25	22	1	ACAGTG	GCGGAC
CBC_MCAV_006	03/23/17	RW	25	24	3	ACAGTG	GTAGCC
CBC_MCAV_007	03/23/17	RW	25	24	2	TTAGGC	GCGGAC
CBC_MCAV_008	03/23/17	RW	25	24	3	ACTTGA	GGCCAC
CBC_MCAV_009	03/23/17	RW	25	24	2	ACAGTG	GGACGG
CBC_MCAV_010	03/23/17	RW	25	24	1	ATCACG	CTCTAC
CBC_MCAV_011	03/23/17	RW	25	28	2	CGATGT	TGACAT
CBC_MCAV_012	03/23/17	RW	25	28	3	ACAGTG	CTCTAC
CBC_MCAV_013	03/23/17	RW	25	28	2	ACAGTG	TTTCAC

CBC_MCAV_014	03/23/17	RW	25	24	1	TGACCA	GTAGCC
CBC_MCAV_015	03/23/17	RW	25	22	3	CGATGT	TGACAT
CBC_MCAV_016	03/23/17	RW	25	24	2	CAGATC	TACAAG
CBC_MCAV_017	03/23/17	RW	16	28	1	TGACCA	CTCTAC
CBC_MCAV_018	03/23/17	RW	16	24	3	ATCACG	GTAGCC
CBC_MCAV_019	03/23/17	RW	16	24	2	CGATGT	GCGGAC
CBC_MCAV_020	03/23/17	RW	16	28	3	ATCACG	CTCTAC
CBC_MCAV_021	03/23/17	RW	16	28	1	ACTTGA	TGACAT
CBC_MCAV_022	03/23/17	RW	16	26	3	CAGATC	TTTCAC
CBC_MCAV_023	03/23/17	RW	16	22	3	ACAGTG	TTGACT
CBC_MCAV_024	03/23/17	RW	16	22	3	CGATGT	GGCCAC
CBC_MCAV_025	03/23/17	RW	16	26	3	CGATGT	TTGACT
CBC_MCAV_026	03/23/17	RW	16	24	1	CGATGT	TACAAG
CBC_MCAV_027	03/23/17	RW	16	24	1	ACAGTG	CTCTAC
CBC_MCAV_028	03/23/17	RW	16	22	3	GCCAAT	GGAACT
CBC_MCAV_029	03/23/17	RW	10	22	1	CAGATC	TTGACT
CBC_MCAV_030	03/23/17	RW	10	24	1	ATCACG	GTAGCC
CBC_MCAV_031	03/23/17	RW	10	24	1	TTAGGC	GTAGCC
CBC_MCAV_032	03/23/17	RW	10	28	1	GCCAAT	GCGGAC
CBC_MCAV_033	03/23/17	RW	10	28	1	GCCAAT	CTCTAC
CBC_MCAV_034	03/23/17	RW	10	28	2	CGATGT	GGACGG
CBC_MCAV_035	03/23/17	RW	10	22	3	TGACCA	TGACAT
CBC_MCAV_036	03/23/17	RW	10	23	2	GCCAAT	TACAAG
CBC_MCAV_037	03/23/17	RW	10	22	3	ACTTGA	TACAAG
CBC_MCAV_038	03/23/17	RW	10	23	3	CGATGT	TTTCAC
CBC_MCAV_039	03/23/17	RW	10	22	2	CGATGT	TTTCAC
CBC_MCAV_040	03/23/17	RW	10	23	2	TGACCA	TTTCAC
CBC_MCAV_041	03/23/17	RW	10	23	3	ACAGTG	TACAAG
CBC_MCAV_042	03/23/17	RW	10	22	3	ACTTGA	GGAACT

CBC_MCAV_043	03/23/17	RW	10	22	2	ATCACG	CTCTAC
CBC_MCAV_044	03/23/17	RW	16	23	1	TTAGGC	TTTCAC
CBC_MCAV_045	03/23/17	RW	16	22	3	ACTTGA	GCGGAC
CBC_MCAV_046	03/23/17	RW	16	22	3	CAGATC	GTAGCC
CBC_MCAV_047	03/24/17	SR	16	22	1	TGACCA	GGCCAC
CBC_MCAV_048	03/24/17	SR	16	22	3	GCCAAT	TGACAT
CBC_MCAV_049	03/24/17	SR	16	23	2	CAGATC	TTGACT
CBC_MCAV_050	03/24/17	SR	16	22	1	GCCAAT	GGAACT
CBC_MCAV_051	03/24/17	SR	16	22	3	GCCAAT	TTTCAC
CBC_MCAV_052	03/24/17	SR	16	26	1	ATCACG	GCGGAC
CBC_MCAV_053	03/24/17	SR	16	23	2	CGATGT	GTAGCC
CBC_MCAV_054	03/24/17	SR	16	24	1	GCCAAT	TTGACT
CBC_MCAV_055	03/24/17	SR	16	22	1	TTAGGC	GGCCAC
CBC_MCAV_056	03/24/17	SR	16	22	2	GCCAAT	GCGGAC
CBC_MCAV_057	03/24/17	SR	16	22	3	GCCAAT	TTGACT
CBC_MCAV_058	03/24/17	SR	16	23	1	ACAGTG	GTAGCC
CBC_MCAV_059	03/24/17	SR	16	22	2	GCCAAT	GGACGG
CBC_MCAV_060	03/24/17	SR	16	23	1	ACTTGA	GGACGG
CBC_MCAV_061	03/24/17	SR	25	22	2	TGACCA	GCGGAC
CBC_MCAV_062	03/24/17	SR	25	28	2	ATCACG	TTTCAC
CBC_MCAV_063	03/24/17	SR	25	22	1	ACAGTG	TGACAT
CBC_MCAV_064	03/24/17	SR	25	23	2	CGATGT	GGCCAC
CBC_MCAV_065	03/24/17	SR	25	23	1	CGATGT	TGACAT
CBC_MCAV_066	03/24/17	SR	25	22	1	TGACCA	TGACAT
CBC_MCAV_067	03/24/17	SR	25	24	3	CAGATC	CTCTAC
CBC_MCAV_068	03/24/17	SR	25	24	1	GCCAAT	TTTCAC
CBC_MCAV_069	03/24/17	SR	25	22	2	ATCACG	GCGGAC
CBC_MCAV_070	03/24/17	SR	25	24	2	GCCAAT	CTCTAC
CBC_MCAV_071	03/24/17	SR	25	24	1	GCCAAT	TACAAG

CBC_MCAV_072	03/24/17	SR	25	24	3	CGATGT	GGACGG
CBC_MCAV_073	03/24/17	SR	25	24	2	ATCACG	TACAAG
CBC_MCAV_074	03/24/17	SR	25	24	2	TTAGGC	GTAGCC
CBC_MCAV_075	03/24/17	SR	25	23	2	CAGATC	GGCCAC
CBC_MCAV_076	03/24/17	SR	10	23	2	ATCACG	TGACAT
CBC_MCAV_077	03/24/17	SR	10	22	3	TTAGGC	TTTCAC
CBC_MCAV_078	03/24/17	SR	10	23	1	ATCACG	TTTCAC
CBC_MCAV_079	03/24/17	SR	10	23	2	ACTTGA	GGACGG
CBC_MCAV_080	03/24/17	SR	10	24	1	TGACCA	TTGACT
CBC_MCAV_081	03/24/17	SR	10	22	1	ACTTGA	GGCCAC
CBC_MCAV_082	03/24/17	SR	10	22	2	TTAGGC	GGCCAC
CBC_MCAV_083	03/24/17	SR	10	22	3	TGACCA	CTCTAC
CBC_MCAV_084	03/24/17	SR	10	22	2	ACAGTG	TGACAT
CBC_MCAV_085	03/24/17	SR	10	22	1	TTAGGC	CTCTAC
CBC_MCAV_086	03/24/17	SR	10	22	1	TGACCA	GGACGG
CBC_MCAV_087	03/24/17	SR	10	22	3	ATCACG	GCGGAC
CBC_MCAV_088	03/24/17	SR	10	22	2	CAGATC	GGACGG
CBC_MCAV_089	03/24/17	SR	10	24	2	CAGATC	TGACAT
CBC_MCAV_090	03/24/17	SR	10	23	1	ACAGTG	GGCCAC
CBC_MCAV_091	03/24/17	SR	10	24	3	TGACCA	GCGGAC
CBC_MCAV_092	03/24/17	SR	16	23	2	ACTTGA	TGACAT
CBC_MCAV_093	03/24/17	TR	16	26	2	TGACCA	CTCTAC
CBC_MCAV_094	03/24/17	TR	16	22	3	CGATGT	GTAGCC
CBC_MCAV_095	03/24/17	TR	16	22	2	GCCAAT	TGACAT
CBC_MCAV_096	03/24/17	TR	16	23	3	GCCAAT	TACAAG
CBC_MCAV_097	03/24/17	TR	16	22	3	ACAGTG	GCGGAC
CBC_MCAV_098	03/24/17	TR	16	24	3	ACAGTG	GGCCAC
CBC_MCAV_099	03/24/17	TR	16	24	1	ATCACG	TTGACT
CBC_MCAV_100	03/24/17	TR	16	22	1	ACTTGA	GGAACT

CBC_MCAV_101	03/24/17	TR	16	22	2	TGACCA	TACAAG
CBC_MCAV_102	03/24/17	TR	16	22	3	TTAGGC	GCGGAC
CBC_MCAV_103	03/24/17	TR	16	22	1	CAGATC	GGAACT
CBC_MCAV_104	03/24/17	TR	16	22	1	GCCAAT	GTAGCC
CBC_MCAV_105	03/24/17	TR	16	22	2	ACTTGA	GGCCAC
CBC_MCAV_106	03/24/17	TR	16	23	1	ATCACG	TGACAT
CBC_MCAV_107	03/25/17	TR	10	22	3	CAGATC	TGACAT
CBC_MCAV_108	03/25/17	TR	10	22	2	ACAGTG	TTGACT
CBC_MCAV_109	03/25/17	TR	10	22	3	CAGATC	GGACGG
CBC_MCAV_110	03/25/17	TR	10	22	1	ACTTGA	TTGACT
CBC_MCAV_111	03/25/17	TR	10	23	3	TGACCA	TTTCAC
CBC_MCAV_112	03/25/17	TR	10	22	1	TTAGGC	TACAAG
CBC_MCAV_113	03/25/17	TR	10	22	2	ATCACG	GTAGCC
CBC_MCAV_114	03/25/17	TR	10	22	2	TGACCA	GGAACT
CBC_MCAV_115	03/27/17	GR	10	22	2	ACAGTG	CTCTAC
CBC_MCAV_116	03/25/17	TR	10	22	1	CAGATC	TTTCAC
CBC_MCAV_117	03/25/17	TR	10	22	1	ACTTGA	GCGGAC
CBC_MCAV_118	03/25/17	TR	10	22	1	CAGATC	GGCCAC
CBC_MCAV_119	03/25/17	TR	10	22	3	ATCACG	TTGACT
CBC_MCAV_120	03/25/17	TR	10	22	1	TGACCA	GGAACT
CBC_MCAV_121	03/25/17	TR	10	22	3	ATCACG	GGAACT
CBC_MCAV_122	03/25/17	TR	25	22	2	TTAGGC	TTGACT
CBC_MCAV_123	03/25/17	TR	25	22	1	TGACCA	TACAAG
CBC_MCAV_124	03/25/17	TR	25	22	1	TGACCA	GCGGAC
CBC_MCAV_125	03/25/17	TR	25	22	1	TTAGGC	GCGGAC
CBC_MCAV_126	03/25/17	TR	25	22	2	ACTTGA	GTAGCC
CBC_MCAV_127	03/25/17	TR	25	22	1	GATCAG	GTAGCC
CBC_MCAV_128	03/25/17	TR	25	22	3	CGATGT	TACAAG
CBC_MCAV_129	03/25/17	TR	25	23	3	ACTTGA	TTGACT

CBC_MCAV_130	03/25/17	TR	25	22	3	ACTTGA	CTCTAC
CBC_MCAV_131	03/25/17	TR	25	22	2	ATCACG	GGAACT
CBC_MCAV_132	03/25/17	TR	25	22	1	TGACCA	TTTCAC
CBC_MCAV_133	03/25/17	TR	25	22	2	ACTTGA	GGAACT
CBC_MCAV_134	03/25/17	TR	25	22	1	CAGATC	TGACAT
CBC_MCAV_135	03/25/17	TR	25	22	3	ACTTGA	GGACGG
CBC_MCAV_136	03/25/17	TR	25	22	3	TGACCA	GGAACT
CBC_MCAV_137	03/27/17	TR	16	22	1	CAGATC	GTAGCC
CBC_MCAV_138	03/27/17	GR	35	23	3	TTAGGC	GGAACT
CBC_MCAV_139	03/27/17	GR	35	22	3	ATCACG	GGACGG
CBC_MCAV_140	03/27/17	GR	35	22	1	ATCACG	GGACGG
CBC_MCAV_141	03/27/17	GR	35	22	3	CAGATC	TTGACT
CBC_MCAV_142	03/27/17	GR	35	22	2	TGACCA	GGACGG
CBC_MCAV_143	03/27/17	GR	35	22	2	CGATGT	TACAAG
CBC_MCAV_144	03/27/17	GR	35	22	1	CAGATC	GCGGAC
CBC_MCAV_145	03/27/17	GR	25	22	3	GCCAAT	CTCTAC
CBC_MCAV_146	03/27/17	GR	25	22	3	TTAGGC	TACAAG
CBC_MCAV_147	03/27/17	GR	25	22	2	TTAGGC	CTCTAC
CBC_MCAV_148	03/27/17	GR	16	22	1	GCCAAT	GGACGG
CBC_MCAV_149	03/27/17	GR	16	26	3	ATCACG	TACAAG
CBC_MCAV_150	03/27/17	GR	16	22	2	CAGATC	TTTCAC
CBC_MCAV_151	03/27/17	GR	16	22	2	CAGATC	GTAGCC
CBC_MCAV_152	03/27/17	GR	10	22	3	ACAGTG	TGACAT
CBC_MCAV_153	03/27/17	GR	10	22	2	GCCAAT	GTAGCC
CBC_MCAV_154	03/25/17	GR	10	22	1	ACTTGA	TTTCAC
CBC_MCAV_155	03/27/17	GR	10	22	2	ACAGTG	GTAGCC
CBC_MCAV_156	03/27/17	TR	10	22	1	CGATGT	GCGGAC
CBC_MCAV_157	03/27/17	GR	10	24	2	ATCACG	GGCCAC
CBC_MCAV_158	03/27/17	GR	10	22	1	ACTTGA	TACAAG

CBC_MCAV_159	03/27/17	GR	10	22	2	ACTTGA	TACAAG
CBC_MCAV_160	03/27/17	GR	10	22	1	CGATGT	GGACGG
CBC_MCAV_161	03/27/17	GR	10	22	2	CGATGT	TTGACT
CBC_MCAV_162	03/27/17	GR	10	22	3	TTAGGC	GTAGCC
CBC_MCAV_163	03/27/17	GR	10	22	3	GCCAAT	GCGGAC
CBC_MCAV_164	03/27/17	GR	10	22	1	TTAGGC	GGAACT
CBC_MCAV_165	03/27/17	GR	10	22	3	ATCACG	TGACAT
CBC_MCAV_166	03/27/17	GR	10	22	1	ATCACG	GGCCAC
CBC_MCAV_167	03/27/17	GR	25	22	2	CGATGT	GGAACT
CBC_MCAV_168	03/27/17	GR	25	22	2	TTAGGC	GGACGG
CBC_MCAV_169	03/27/17	GR	25	24	2	CAGATC	CTCTAC
CBC_MCAV_170	03/27/17	GR	25	23	2	ACAGTG	GGAACT
CBC_MCAV_171	03/27/17	GR	25	24	1	CGATGT	GTAGCC
CBC_MCAV_172	03/27/17	GR	25	22	3	CAGATC	GGCCAC
CBC_MCAV_173	03/27/17	GR	25	22	2	ATCACG	GGACGG
CBC_MCAV_174	03/27/17	GR	25	22	2	ACAGTG	GCGGAC
CBC_MCAV_175	03/27/17	GR	25	24	2	TGACCA	GTAGCC
CBC_MCAV_176	03/27/17	GR	16	22	3	CGATGT	CTCTAC
CBC_MCAV_177	03/27/17	GR	16	22	3	ACTTGA	GTAGCC
CBC_MCAV_178	03/27/17	GR	16	24	1	ACAGTG	GGACGG
CBC_MCAV_179	03/27/17	GR	16	23	1	ACAGTG	TTGACT
CBC_MCAV_180	03/27/17	GR	16	28	2	ACAGTG	GGCCAC
CBC_MCAV_181	03/27/17	GR	16	22	3	TTAGGC	TTGACT
CBC_MCAV_182	03/27/17	GR	16	22	1	ACTTGA	CTCTAC
CBC_MCAV_183	03/27/17	GR	16	24	2	ACTTGA	CTCTAC
CBC_MCAV_184	03/27/17	GR	25	22	2	TTAGGC	TACAAG
CBC_MCAV_185	03/27/17	GR	25	24	1	CAGATC	TACAAG
CBC_MCAV_186	03/27/17	GR	25	24	2	TTAGGC	TGACAT
CBC_MCAV_187	03/27/17	GR	16	22	3	ATCACG	TTTCAC

CBC_MCAV_188	03/27/17	GR	16	22	3	TGACCA	TTGACT
CBC_MCAV_189	03/27/17	GR	16	24	3	TGACCA	GGCCAC
CBC_MCAV_190	03/27/17	GR	35	23	2	TGACCA	TTGACT
CBC_MCAV_191	03/27/17	GR	35	22	2	CAGATC	GCGGAC
CBC_MCAV_192	03/27/17	GR	35	22	2	TGACCA	TGACAT
CBC_MCAV_193	03/27/17	GR	35	22	2	ACTTGA	GCGGAC
CBC_MCAV_194	03/27/17	GR	35	22	3	CAGATC	TACAAG
CBC_MCAV_195	03/27/17	GR	35	22	3	CGATGT	GCGGAC
CBC_MCAV_196	03/27/17	GR	35	22	2	GCCAAT	GGAACT
CBC_MCAV_197	03/05/16	GR	35	22	1	CGATGT	TTTCAC
CBC_MCAV_198	03/05/16	SR	35	22	2	ACTTGA	TTGACT
CBC_MCAV_199	03/05/16	SR	35	24	3	ATCACG	GGCCAC
CBC_MCAV_200	03/05/16	SR	35	24	3	TTAGGC	GGCCAC
CBC_MCAV_201	03/05/16	SR	35	24	1	GCCAAT	TGACAT
CBC_MCAV_202	03/05/16	SR	35	28	2	GCCAAT	TTTCAC
CBC_MCAV_203	03/05/16	SR	35	22	3	ACAGTG	GGACGG
CBC_MCAV_204	03/05/16	SR	35	22	2	ACAGTG	TACAAG
CBC_MCAV_205	03/05/16	SR	35	24	2	TTAGGC	TTTCAC
CBC_MCAV_206	03/05/16	SR	35	24	2	ATCACG	TTGACT
CBC_MCAV_207	03/06/16	SR	35	24	1	ACAGTG	GGAACT
CBC_MCAV_208	03/05/16	SR	35	24	2	TGACCA	GGCCAC
CBC_MCAV_209	03/05/16	SR	35	28	3	GCCAAT	GGCCAC
CBC_MCAV_210	03/05/16	SR	35	23	3	TGACCA	GTAGCC
CBC_MCAV_211	03/06/16	RW	35	26	3	TGACCA	TACAAG
CBC_MCAV_212	03/06/16	RW	35	28	3	TTAGGC	CTCTAC
CBC_MCAV_213	03/06/16	RW	35	26	1	ATCACG	GGAACT
CBC_MCAV_214	03/06/16	RW	35	24	2	CGATGT	CTCTAC
CBC_MCAV_215	03/06/16	RW	35	28	3	CAGATC	GGAACT
CBC_MCAV_216	03/06/16	RW	35	28	1	ACAGTG	TACAAG

CBC_MCAV_217	03/06/16	RW	35	28	1	CGATGT	CTCTAC
CBC_MCAV_218	03/06/16	RW	35	22	1	GCCAAT	GGCCAC
CBC_MCAV_219	03/06/16	RW	35	28	3	TTAGGC	TGACAT
CBC_MCAV_220	03/06/16	RW	35	28	1	CAGATC	CTCTAC
CBC_MCAV_221	03/06/16	RW	35	27	2	GCCAAT	TTGACT
CBC_MCAV_222	03/06/16	RW	35	28	1	CGATGT	GGCCAC
CBC_MCAV_223	03/07/16	RW	35	28	1	TTAGGC	TGACAT
CBC_MCAV_224	03/06/16	RW	35	28	3	ACTTGA	TGACAT
CBC_MCAV_225	03/06/16	RW	35	28	3	ACAGTG	TTTCAC
CBC_MCAV_226	03/07/16	TR	35	28	3	TGACCA	GGACGG
CBC_MCAV_227	03/07/16	TR	35	28	1	TTAGGC	GGACGG
CBC_MCAV_228	03/07/16	TR	35	26	1	ACAGTG	TTTCAC
CBC_MCAV_229	03/07/16	TR	35	28	3	GCCAAT	GGACGG
CBC_MCAV_230	03/07/16	TR	35	22	3	ACAGTG	GGAACT
CBC_MCAV_231	03/08/16	TR	35	28	1	CAGATC	GGACGG
CBC_MCAV_232	03/08/16	TR	35	25	2	GCCAAT	GGCCAC
CBC_MCAV_233	03/25/17	TR	35	28	1	TTAGGC	TTGACT
CBC_MCAV_234	03/08/16	TR	35	28	3	ACTTGA	TTTCAC
CBC_MCAV_235	03/08/16	TR	35	N/A	N/A	N/A	N/A
CBC_MCAV_236	03/08/16	TR	35	24	2	TTAGGC	GGAACT
CBC_MCAV_237	03/08/16	TR	35	24	3	GCCAAT	GTAGCC
CBC_MCAV_238	03/08/16	TR	35	24	1	ATCACG	TACAAG
CBC_MCAV_239	03/08/16	TR	35	24	2	CAGATC	GGAACT
CBC_MCAV_240	03/08/16	TR	35	24	1	ACTTGA	GTAGCC
CBC_MCAV_241	03/08/16	SR	35	24	3	CGATGT	GGAACT
CBC_MCAV_242	03/08/16	SR	35	24	3	TTAGGC	GGACGG

Supplementary Table S3. Test results from permutational multivariate analysis of variance (PERMANOVA; 9,999 permutations) of Symbiodiniaceae *ITS2* type profiles from *M. cavernosa* colonies and pairwise comparisons between all depth zones (FDR corrected) without 35 m samples. Non-significant tests are listed as “ns”.

**Test**	**Comparison**	**Pseudo-*F***	***p*-value**
Overall	Depth	5.6348	0.0002
	Site	1.4764	ns
	Depth:Site	1.2106	ns
Depth	10 m vs 16 m	0.9838	ns
	10 m vs 25 m	9.0844	0.0006
	16 m vs 25 m	9.4051	0.0006
